# Evaluation of rare *NR1D2* variants in MODY-X: clinical, genetic, and in silico insights

**DOI:** 10.1007/s40618-026-02821-7

**Published:** 2026-02-06

**Authors:** Cagatay Aydogan, Deniz Kanca-Demirci, Nurdan Gul, Sukran Poyrazoglu, Bengu Tokat, Ummu Mutlu, Oguz Ozturk, Hulya Yilmaz-Aydogan, Ilhan Satman

**Affiliations:** 1 Department of Molecular Medicine, Aziz Sancar Institute of Experimental Medicine, Istanbul University, Istanbul, Turkey; 2 Department of Molecular Biology and Genetics, Faculty of Arts and Sciences, Halic University, Istanbul, Turkey; 3 Istanbul Faculty of Medicine, Division of Endocrinology and Metabolism, Department of Internal Medicine, Istanbul University, Istanbul, Turkey; 4 Pediatric Endocrinology Unit, Department of Pediatrics, Istanbul Faculty of Medicine, Istanbul University, Istanbul, Turkey; 5 Department of Genetics and Genomic Sciences, Icahn School of Medicine at Mount Sinai, New York, USA

**Keywords:** Diabetes, MODY, NR1D2, Circadian rhythm

## Abstract

**Purpose:**

The objective of this study was to investigate the protein-coding regions of the NR1D2 gene in patients clinically diagnosed with maturity-onset diabetes of the young (MODY), including those without pathogenic variants in known MODY genes (MODY-X), and to characterize the potential functional relevance of detected variants.

**Methods:**

The variants present in the exons and adjacent intronic regions of the *NR1D2* gene in patients with MODY were subjected to comparative analysis with those observed in healthy individuals and patients with type 2 diabetes mellitus. The maximum credible allele frequency was set to be 0.0001. The potential impact of rare variants was evaluated using variety in silico prediction tools, including PolyPhen-2, SIFT, MutationTaster2025, FATHMM-XF, REVEL, CADD, and DynaMut2.

**Results:**

Two extremely rare NR1D2 missense variants were identified in three MODY-X patients: p.I148V (rs148928938) in exon 4 and p.R286W (rs768518229) in exon 5, with allele frequencies of 74 per million and 3 per million in gnomAD, respectively. In silico predictions indicated a more consistent deleterious profile for p.I148V, whereas p.R286W demonstrated heterogeneous and predominantly benign or borderline predictions. The clinical manifestations exhibited by the carriers were found to be variable, which is consistent with the metabolic heterogeneity that is characteristic of MODY.

**Conclusion:**

The findings suggest that these variants may function as metabolic modifiers contributing to phenotypic variability in MODY-X. Prospective family-based studies and functional assays are needed to clarify their biological significance.

**Supplementary Information:**

The online version contains supplementary material available at 10.1007/s40618-026-02821-7.

## Introduction

Maturity-onset diabetes of the young (MODY) is a type of diabetes characterized by the absence of autoimmune features, predominantly genetic, and monogenic, diagnosed in non-obese young adults [1]. Patients are typically diagnosed before the age of 25, although the onset of MODY can sometimes be delayed until age 40 [2]. In most cases of MODY, causal variants have been identified in genes encoding the *glucokinase enzyme* (*GCK*) and three transcription factors: *hepatocyte nuclear factor (HNF)−4*α (*HNF4A*), *−1α (HNF1A)*, and *− 1β* (*HNF1B*) [2, 3]. While evidence refuting the association between *BLK*, *KLF11*, and *PAX4* with MODY is increasing, mutations in over 10 genes are known to cause MODY [3, 4]. However, variants in known genes cannot explain all cases of MODY, particularly in some populations [5, 6]. Additionally, variants in genes other than *GCK* typically have low penetrance [7]. Therefore, in addition to investigating additional causal genes, epigenetic effects, and factors that may delay the onset of the disease, it is also crucial to study factors that explain variable penetrance in MODY.

Nuclear receptors are transcription factors that respond to metabolic ligands and regulate the transcription of numerous genes that are involved in various processes within biological processes, such as cell proliferation, development, metabolism, and reproduction [8, 9]. The nuclear receptor subfamily 1 group D (NR1D), also known as REVERB, is a family consisting of NR1D1 and NR1D2 [10]. NR1Ds have been demonstrated to play a pivotal role in a number of significant physiological processes, ranging from the regulation of the circadian rhythm to the maintenance of lipid homeostasis [11]. It has been established that both NR1D1 (REVERB-α or REVERB-A) and NR1D2 (REVERB-β or REVERB-B) play a vital role in regulating circadian rhythms in the brain and peripheral tissues by directly targeting genes such as *BMAL1* and *CLOCK* [12]. The components of the circadian clock have been demonstrated to regulate glucose and lipid homeostasis by controlling the activities and expression of related enzymes [13]. Furthermore, the circadian clock is imperative for sustaining the body’s physiological functions. It has been demonstrated to regulate various cyclic activities, including hormone secretion, body temperature regulation, insulin sensitivity, and sleep-wake patterns [14]. Recent studies have indicated that the presence of specific variants in the *NR1Ds* locus is associated with alterations in the levels of certain metabolites, including serum high-density lipoprotein-cholesterol (HDL-C) and albumin, in patients with type 2 diabetes mellitus (T2DM) [15]. Consequently, the function of NR1Ds and other elements of the circadian rhythm in metabolic diseases is a promising area for further research. In this study, variants in the exons and intronic regions near exons in the *NR1D2* gene of patients clinically diagnosed with MODY but classified as “MODY-X” due to the absence of causal gene mutations in known MODY genes were investigated by comparing them with those of the healthy control and T2DM groups. Subsequently, the potential for variants that meet specific criteria to induce the disease state was assessed by employing in silico tools.

## Research design and methods

### Patient selection

The present study comprised 79 control samples, 75 MODY patients and 66 T2DM patients, who had previously participated in the MODY-IST study at Istanbul University. These patients had consulted paediatric and adult endocrinology clinics. In the MODY group, mutations in genes associated with MODY were confirmed in 27 patients, while 19 clinically MODY patients without at least one causal mutation in known MODY genes were classified as MODY-X [16, 17]. In patients diagnosed with MODY, specific clinical characteristics were identified, including the following: the age-at-onset of diabetes, the presence of ongoing endogenous insulin production, a family history of diabetes extending over at least three generations, the absence of pancreatic autoantibodies (e.g., islet-cell cytoplasmic (ICA), islet antigen-2 (IA-2 A), and glutamic acid decarboxylase (GADA)), and the absence of ketoacidosis. The control group consisted of healthy individuals without any metabolic disorders and without a family history of diabetes in first-degree relatives, and they did not have any MODY-specific mutations. T2DM patients were diagnosed according to the current criteria (1). Informed consent, in writing, was obtained from all individuals or their parents or guardians. Homeostatic model assessment of insulin resistance (HOMA-IR) formula was used to evaluate insulin resistance *(fasting serum insulin (mIU/L) × fasting plasma glucose (mg/dL)/405)* [18]. The study design was approved by Ethics Committee of Istanbul Faculty of Medicine (Approval no. 2014/922) and complied with the Declaration of Helsinki for medical research involving human subjects.

## Exome sequencing

DNA isolation was performed using the Epicenter MasterPure DNA purification kit (Lucigen, Wisconsin, USA) from peripheral blood samples. After verifying the purity and concentration of DNA spectrophotometrically using the NanoDrop 2000c (Thermo Fisher, Massachusetts, USA), a long polymerase chain reaction was performed on the exons and exon-intron boundaries of the *NR1D2* gene in a C1000 thermal cycler (Bio-Rad, California, USA). Libraries were constructed using the TruSeq Custom Amplicon v1.5 Exome Library Prep kit (Illumina, California, USA). Exome sequencing and paired-end sequencing of the libraries were performed using the MiSeq 4000 system (Illumina).

## Bioinformatic analysis

In this study, variants found in the exons and adjacent intron regions of the *NR1D2* gene in MODY patients were subjected to comparative analysis with those observed in healthy individuals and T2DM patients. By considering the low genetic heterogeneity and potentially low penetrance of these variants in MODY, a maximum allele frequency of 0.0001 was utilised to reduce the impact of validation bias. Subsequently, the frequencies of the existing alleles were checked in gnomAD (Genome Aggregation Database) v4.1.0, and alleles with a frequency above 0.0001 were excluded from the study [19]. The remaining mutations were then subjected to a comprehensive in silico evaluation. To assess protein-level functional and structure consequences, SIFT (Sorting Intolerant From Tolerant) [20], Polyphen-2 (Polymorphism Phenotyping v2) [21], FATHMM-XF [22], and DynaMut2 [23] were employed, as these tools directly model the potential impact of amino acid substitutions on protein function or stability. Additionally, broader pathogenicity predictors including MutationT@ster2025 [24], REVEL (Rare Exome Variant Ensemble Learner) [25], and CADD (Combined Annotation Dependent Depletion) [26] tools were used to integrate evolutionary, biochemical, and multi-feature information. Each variant was also queried in the ClinVar database to determine previously reported clinical significance [27].

## Results

### Clinical and metabolic characteristics of the MODY-X cohort

The MODY-X cohort exhibited clinical features consistent with early-onset, non-insulin-dependent diabetes. The mean age of the patients and the mean age at diabetes onset were 31.6 ± 19.3 years and 19.8 ± 11.7 years, respectively. All MODY-X patients exhibited a positive family history of diabetes, indicative of a typical strong genetic background. Anthropometric and metabolic parameters exhibited substantial variability among MODY-X patients. The median body mass index (BMI) was 25.6 kg/m², indicating that a proportion of cases exhibited mild degrees of overweight. The markers of glycemic control exhibited significant increase (mean fasting plasma glucose (FPG) 165.7 ± 70.5 mg/dL and glycosylated hemoglobin A1c (HbA1c) (%) 7.48 ± 1.79%). The study found that 36.8% (*n* = 7) of the patient population exhibited a HOMA-IR score above the reference value of 2.5, indicative of insulin resistance. The lipid profile exhibited a heterogeneous distribution, manifesting a dyslipidemic pattern in some patients. Thyroid function tests and autoantibodies were largely within reference limits. Liver and kidney functions were preserved in most cases, and diabetes-related complications were relatively low (Table 1).

**Table 1:** Characteristics of the MODY-X group

	*n*	%	Reference values
**Demographic**			
Gender (female/male)	12/7	63/37	
Family history of diabetes (yes/no)	19/0	100/0	
	**X ± SD**	**Median (IQR)**	
Age (year)	31.6 ± 19.3	41 (14.5)	
Age-at-onset of diabetes (year)	19.8 ± 11.7	24 (15.5)	
Body mass index at diagnosis (kg/m^2^)	25.6 ± 5.9	28.5 (3.5)	< 25
Systolic blood pressure (mmHg)	118.5 ± 5.5	120 (7.5)	< 120
Diastolic blood pressure (mmHg)	74.7 ± 8.6	80 (5)	< 80
**Glycemic markers**			
Fasting plasma glucose (mg/dL)	165.7 ± 70.5	196 (99)	70–100
Glycosylated hemoglobin A1c (%)	7.48 ± 1.79	8.5 (3.1)	4–5.6
C-peptide (ng/mL)	1.94 ± 0.90	1.17 (1.34)	1.1–4.4
HOMA-IR	4.78 ± 1.58	1.57 (1.45)	< 2.5
**Lipid profile**			
Triglycerides (mg/dL)	135.5 ± 99.1	157 (282.5)	< 150
High-density lipoprotein-cholesterol (mg/dL)	47.5 ± 15.5	43 (23.5)	> 40
Low-density lipoprotein-cholesterol (mg/dL)	109.1 ± 31.7	120 (29.5)	< 130
**Thyroid function**			
Thyroid stimulating hormone (mIU/L)	2.37 ± 1.35	1.68 (3.04)	0.27–4.2
Free thyroxine (ng/dL)	15.79 ± 2.06	16. 08 (1.02)	12–22
Anti-thyroid peroxidase antibody (IU/mL)	12.9 ± 12.7	9.0 (30.8)	< 35
Anti-thyroglobulin antibody (IU/mL)	67.3 ± 53.5	25.8 (137.5)	< 115
**Liver function**			
Alanine aminotransaminase (U/L)	30.6 ± 20.7	22 (41)	5–45
Aspartate aminotransaminase (U/L)	24.6 ± 12.9	40 (26)	5–42
Gamma glutamyl transferase (U/L)	39.1 ± 31.9	69 (63)	5–85
High-sensitivity C-reactive protein (mg/L)	2.91 ± 2.80	1.01 (5.08)	< 5
**Kidney function**			
Uric acid (mg/dL)	4.2 ± 1.5	4.2 (3.4)	2.5–7.5
Blood urea nitrogen (mg/dL)	14.3 ± 6.4	15 (10.5)	8–22
Urea (mg/dL)	25.7 ± 11.3	15 (26.5)	0–50
Creatinine (mg/dL)	0.71 ± 0.21	0.8 (0.32)	0.7–1.4
Estimated glomerular filtration rate (mL/min./1.73 m^2^)	109.2 ± 31.6	110 (33.5)	> 90
Urine albumin-to-creatinine ratio (mg/g)	13.3 ± 6.7	3.3 (6.1)	< 30
**Diabetes-related complications**	**n**	**%**	
Nephropathy (yes/no)	2/17	10.5/89.5	
Neuropathy (yes/no)	4/15	21.1/78.9	
Retinopathy (yes/no)	2/17	10.5/89.5	

Categorical variables were presented as sample size (n) and percentage (%), while continuous variables were presented as mean (± standard deviation) and median (interquartile range)

MODY-X, maturity-onset diabetes of the young patients with no confirmed genetic causes; HOMA-IR, homeostatic model assessment of insulin resistance

### Genetic analysis

#### Sequence variants in *NR1D2* gene

Following a comprehensive evaluation of the relevant criteria, it was determined that adenine > guanine and cytosine > thymine missense mutations were identified in two distinct exons of three MODY-X patients. The initial missense mutation identified is rs148928938, located in exon 4, which results in the 148th amino acid being substituted from isoleucine to valine (both of which are branched-chain amino acids). The other mutation, rs768518229, located in exon 5, results in the replacement of the basic arginine residue at position 286 with the hydrophobic and aromatic tryptophan (Table 2). According to the data contained in the gnomAD resource, which comprises information on over 1.5 million alleles, the frequencies of these missense mutations were determined to be 74 and 3 per million, respectively [19]. NR1D2 mutations were not detected in MODY patients previously identified with mutations in MODY-associated genes, in the healthy control group, or in the T2DM groups [16, 17].

**Table 2:** In Silico pathogenicity predictions for the *NR1D2* p.I148V and p.R286W variants across multiple computational platforms

NR1D2 mutation (GRCh38.p14)	NR1D2 *p*.Ile148Val		NR1D2 *p*.Arg286Trp	
Chromosomal position	chr3: 23,959,740		chr3: 23,962,315	
Rs no	rs148928938		rs768518229	
Reference allele	A		C	
Minor allele	G		T	
gnomAD Allele Frequency (v4.1.0)	G = 0.00007373		T = 0.000003098	
Transcript variant	NM_001145425.2:c.217 A > C		NM_001145425.2:c.631 C > T	

In Silico Predictors	Score/Prediction	Interpretation	Score/Prediction	Interpretation
Polyphen	0.954	Probably damaging	0.014	Benign
SIFT	0.05	Deleterious (at threshold)	0.06	Tolarated (borderline)
MutationT@ster2025	Deleterious	Disease causing	Deleterious	Disease causing
FATHMM-XF	0.737876	Damaging	0.154967	Benign
REVEL	0.661	Likely pathogenic	0.501	Uncertain
CADD Phred	23	Deleterious	20.6	Deleterious
DynaMut2 (ΔΔG)	–1.76 kcal/mol	Destabilizing	–0.58 kcal/mol	Mild destabilizing

#### In silico analysis

The potential functional impact of the *NR1D2* missense variants p.I148V and p.R286W was assessed using a comprehensive multi-platform in silico prediction strategy (Table 2). For p.I148V, several tools predicted a damaging or destabilizing effect. PolyPhen-2 indicated a likely damaging impact (0.954), SIFT classified the substitution as deleterious at the decision threshold (0.05), and MutationTaster2025 identified the variant as disease-causing. FATHMM-XF also supported a damaging effect (0.737876), while REVEL yielded a likely pathogenic score (0.661). The CADD score (23) placed the variant within the deleterious range, and DynaMut2 predicted a notable reduction in protein stability (ΔΔG = − 1.76 kcal/mol). For p.R286W, the in silico predictions were more heterogeneous. PolyPhen-2 suggested a benign effect (0.014), and FATHMM-XF exhibited a similarly benign tendency (0.154967). The SIFT yielded a borderline tolerated value (0.06), whereas MutationTaster2025 classified the variant as disease-causing. The REVEL generated an uncertain score (0.501), and the CADD value (20.6) fell near the lower end of the deleterious spectrum. The DynaMut2 model predicted only mild structural destabilization (ΔΔG = − 0.58 kcal/mol). However, no pathogenicity data is available for these mutations in ClinVar. Taken together, these results indicate that p.I148V is supported by a more consistent deleterious signature across multiple prediction algorithms, whereas p.R286W exhibits a mixed profile with predominantly benign or borderline outputs. This pattern is consistent with a potential low-to-moderate effect model rather than a uniformly pathogenic impact.

#### Pedigree analyses of the three *NR1D2* mutation–positive patients

**Fig. 1:**
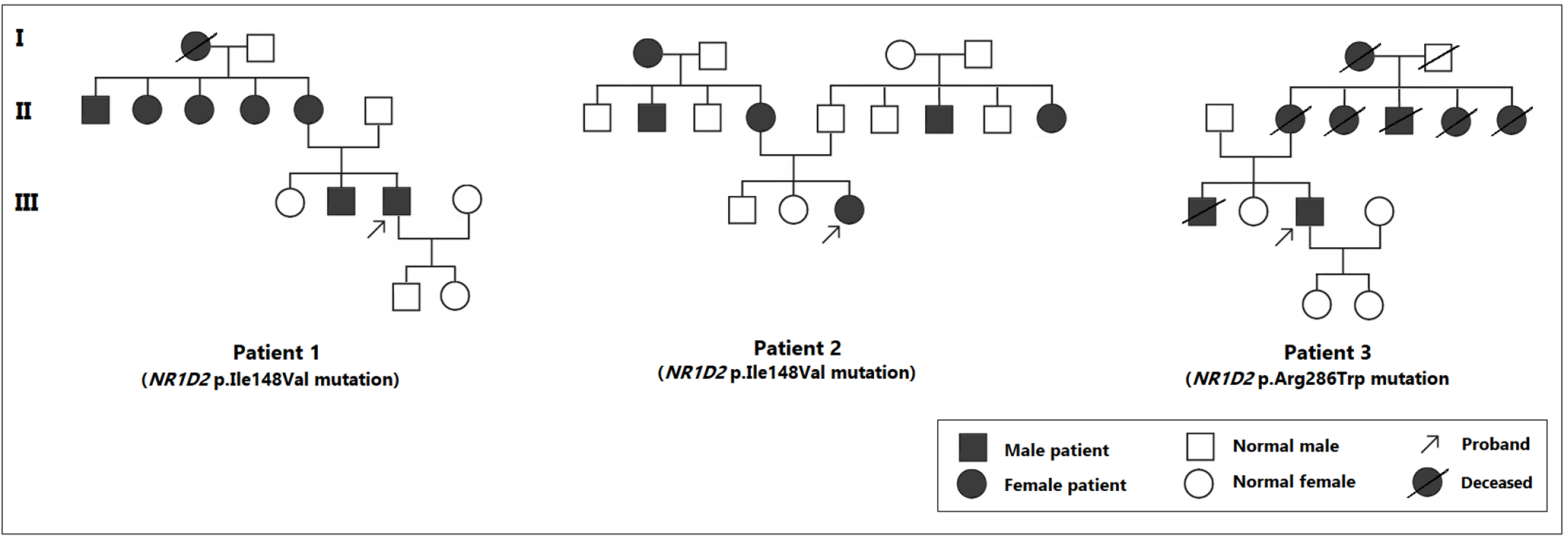
The pedigree structures of three MODY-X probands carrying the *NR1D2* missense variants. The probands are indicated by arrows. In all families, diabetes shows vertical transmission across at least three generations, consistent with an autosomal dominant inheritance pattern. Genetic testing was performed only in the probands; therefore, the *NR1D2* variant status of affected relatives remains unknown

Pedigree evaluation revealed multigenerational transmission of diabetes in all *NR1D2* variant–positive families (Fig. 1), reflecting a pattern concordant with autosomal dominant inheritance. Despite the inability to conduct segregation analysis due to the retrospective study design and the unavailability of family genetic samples, the recurrence of diabetes across multiple generations offers compelling evidence supporting a shared genetic predisposition among these relatives.

#### Clinical and metabolic characteristics of *NR1D2* mutation carriers

**Patient 1** (male, 41-year-old) carried the *NR1D2* p.I148V mutation. He was diagnosed with early-onset diabetes (FPG 100 mg/dL, HbA1c 8.5%). The BMI was overweight (28.5 kg/m²). The patient’s lipid profile revealed a hypertriglyceridemia (448 mg/dL) and low HDL-C (33 mg/dL), indicating dyslipidemia. The elevated thyroid autoantibodies against thyroid peroxidase and thyroglobulin (anti-TPO: 55.6 IU/mL; anti-Tg: 261 IU/mL) were identified, while thyroid function was within normal ranges. The kidney and liver parameters were within reference limits.

**Patient 2** (female, 23-year-old) also carried the p.I148V mutation. The patient first presented with prediabetes (FPG: 110 mg/dL and HbA1c: 5.7%), soon after turned to mild diabetes accompanied by an elevated BMI of 26.7 kg/m². In contrast to Patient 1, Patient 2 exhibited a normal fasting lipid profile and no autoimmune thyroiditis.

**Patient 3** (male, 58-year-old) with the *NR1D2* p.R286W mutation presented with moderate hyperglycemia (FPG 144 mg/dL, HbA1c 7.3%) and hypertension. The BMI was normal (23.9 kg/m²). While fasting triglycerides was within normal limits, low-density lipoprotein cholesterol (LDL-C) was moderately elevated (151 mg/dL) despite the administration of statin therapy. Serum anti-Tg was high, yet thyroid hormones were within the normal range.

During follow-up, none of these patients developed any microvascular complications. All three maintained normal β-cell reserve. In Patient 1 and Patient 3, lipid levels remained high despite anti-lipid therapy. The thyroid ultrasound images revealed heterogeneous parenchymal tissue, and a diagnosis of Hashimoto’s thyroiditis was made. However, as thyroid hormone levels remained normal, they did not require L-thyroxine replacement therapy. Clinical and laboratory data of patients with *NR1D2* mutations detected at the time of genetic testing are shown in Table 3.

**Table 3:** The clinical characteristics of MODY-X patients with *NR1D2* gene mutation

	Patient 1	Patient 2	Patient 3
**Patient code**	DAH1	DAH2	DAH56
*NR1D2* mutation	p.Ile148Val (rs148928938)	p.Ile148Val (rs148928938)	p.Arg286Trp (rs768518229)
**Demographic**			
Gender	Male	Female	Male
Age (year)	41	23	58
Age at diagnosis of diabetes (year)	34	16	38
Body mass index at diagnosis (kg/m^2^)	28.5	26.7	23.9
**Glycemic markers**			
Fasting plasma glucose (mg/dL)	100	110	144
Glycosylated hemoglobin A1c (%)	8.5	5.7	7.3
C-peptide (ng/mL)	1.99	1.69	1.91
HOMA-IR	1.57	1.57	1.60
**Lipid profile**			
Triglycerides (mg/dL)	448	115	91
High-density lipoprotein-cholesterol (mg/dL)	33	59	43
Low-density lipoprotein-cholesterol (mg/dL)	128	127	151
**Thyroid function**			
Thyroid stimulating hormone (mIU/L)	2.07	1.45	3.54
Free thyroxine (ng/dL)	16.4	11.7	14.7
Anti-thyroid peroxidase antibody (IU/mL)	55.6	7	9.6
Anti-thyroglobulin antibody (IU/mL)	261	15	223
**Liver function**			
Alanine aminotransaminase (U/L)	19	9	18
Aspartate aminotransaminase (U/L)	14	10	21
Gamma glutamyl transferase (U/L)	18	4	22
High-sensitivity C-reactive protein (mg/L)	0.40	1.28	3.57
**Kidney function**			
Uric acid (mg/dL)	5.3	1.3	4.2
Blood urea nitrogen (mg/dL)	15	3	21
Urea (mg/dL)	15	5	42
Creatinine (mg/dL)	0.8	0.7	0.8
Estimated glomerular filtration rate (mL/min./1.73 m^2^)	110	161	98.5
Urine albumin-to-creatinine ratio (mg/g)	12.8	19	8.27
**Diabetes-related complications**			
Nephropathy (Yes/No)	No	No	No
Neuropathy (Yes/No)	No	No	No
Retinopathy (Yes/No)	No	No	No
Other chronic diseases	Hashimoto thyroiditis	No	Hypercholesterolemia, Hashimoto thyroiditis, Hypertension
**Medications**	Insulin, Metformin	Insulin	SU, Insulin, Statin, ARB, Thiazide

HOMA-IR, homeostatic model assessment of insulin resistance; SU, sulfonylurea; ARB, angiotensin-II receptor blocker

## Discussion

MODY is a dominantly inherited familial disease, but it is clinically highly heterogeneous and often misdiagnosed [1, 28]. Diagnosing and genetically typing MODY can help differentiate it from other types of diabetes, provide clearer statistics on MODY, and facilitate more targeted and personalized treatments [28, 29]. Further research is required to provide a more precise definition of the etiologies of MODY genes and the characteristics and penetrance of causal variants in these genes, thereby enabling more precise clinical decisions [2, 3]. Furthermore, given the low penetrance of MODY variants and their non-dominant inheritance characteristics, the categorisation of certain genes as MODY genes is being revised. Consequently, these genes are no longer recommended for inclusion in MODY genetic testing, and the genotype-phenotype relationship is not always direct. Consequently, there is a growing imperative to investigate the effects of other genes, epigenetic and environmental factors, and potential variants in non-pathogenic *cis* and *trans* genetic regulatory elements that may delay MODY [4, 30].

**Fig. 2:**
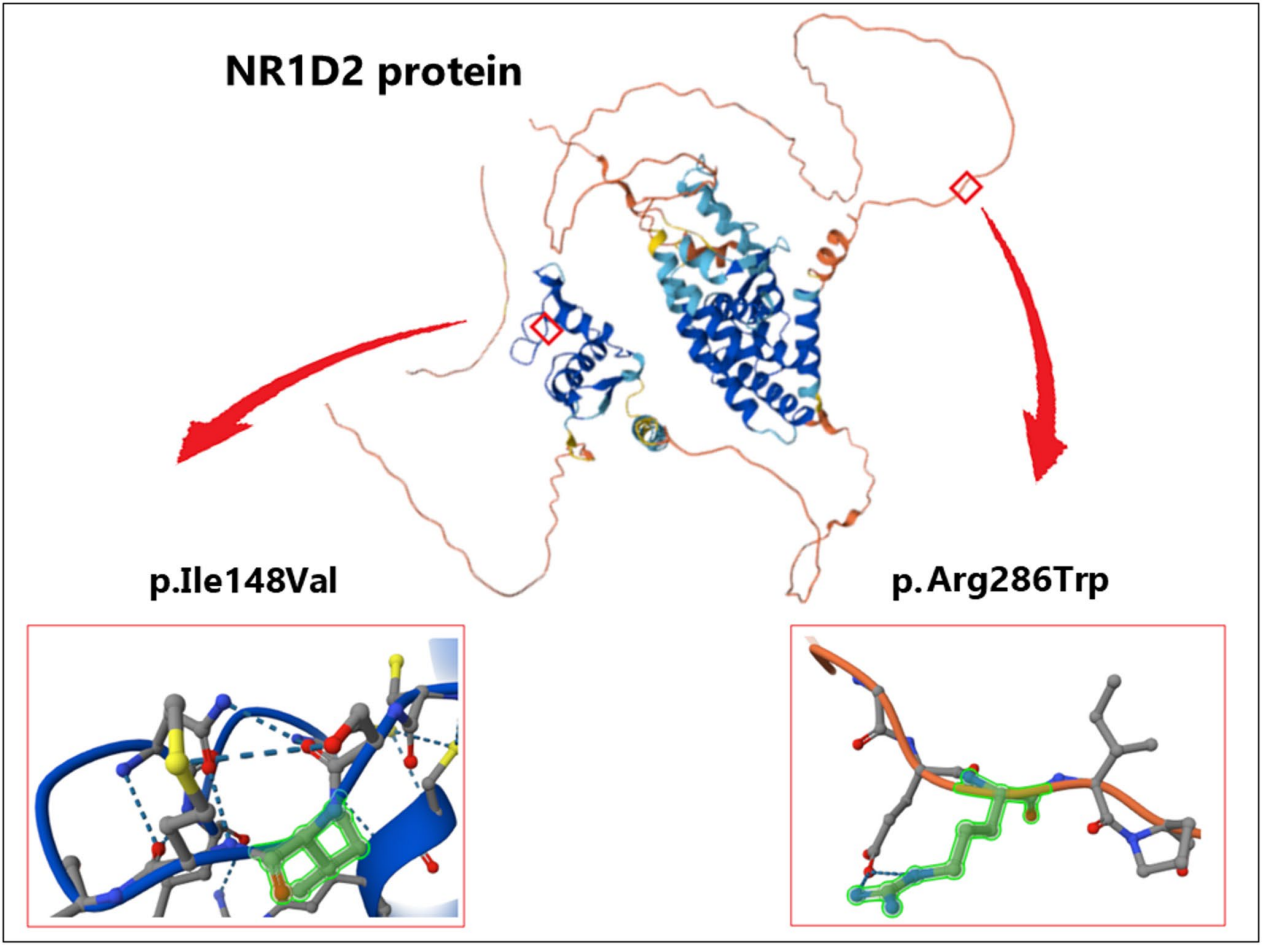
Structural localization of *NR1D2* missense variants p.I148V and p.R286W within the protein model. Structural models and residue-level visualizations were generated using ProtVar (UniProt/AlphaFold)

In this study, variations in the exons and adjacent intron regions of the *NR1D2* gene were examined in patients clinically diagnosed with MODY, both those with and without mutations (MODY-X) identified in known MODY genes. Furthermore, the potential of certain variants to cause MODY was assessed using computational tools. Two *NR1D2* missense mutations (p.I148V (rs148928938) and p.R286W (rs768518229)), located in separate exons, were identified in three patients with MODY-X. It is notable that *NR1D2* mutations were not detected in 27 MODY patients with previously identified mutations in the MODY-IST Study, in genes associated with MODY, nor in healthy controls and T2DM groups [16, 17]. The initial mutation, designated as rs148928938, is located within exon 4 and leads to a substitution of Ile with Val at position 148. This amino acid substitution is located within the DNA binding domain (Fig. 2), which exhibits a highly analogous protein sequence in *NR1Ds* [11]. The second mutation, rs768518229, located in exon 5 of the *NR1D2* gene, results in a substitution from Arg to Trp at position 286. This mutation at position Arg286, is located in the region between the DNA binding and ligand binding domains, known as the hinge region of nuclear receptors (Fig. 2) [11]. In silico analyses of the p.I148V and p.R286W variants yielded partially discordant results, thus illustrating the inherent limitations of relying on individual prediction tools for pathogenicity assessment. While multiple algorithms indicated deleterious or destabilizing effects for p.I148V, p.R286W showed predominantly benign or borderline outcomes. This heterogeneity is consistent with variants that exert modest or context-dependent effects, rather than the uniform signatures expected for high-penetrance monogenic mutations. While both variants are present in gnomAD at a low frequency, this finding alone does not substantiate a pathogenic role in the absence of segregation or functional data. These findings support the interpretation of the *NR1D2* variants as biologically plausible modifiers rather than definitive causative alleles.

Increasing evidence suggests that circadian dysfunction is associated with increased insulin resistance and metabolic irregularities [31]. The nuclear receptor NR1D2 has emerged as a promising therapeutic target due to its critical involvement in various biological processes, including circadian rhythms, inflammation, tumor formation, and glucose and lipid metabolism [11]. However, further research is necessary to elucidate the underlying mechanisms. Since NR1D1 and NR1D2 exhibit overlapping tissue distribution and partially redundant transcription functions, abnormalities in one paralog may alter compensatory mechanisms of the other. Importantly, *NR1D1/NR1D2* expression is known to increase in an age-dependent and male-specific manner [32], suggesting that metabolic traits may be differentially modulated by these transcription factors depending on sex and age. Clock gene polymorphisms exhibit associations with clinical markers of noncommunicable diseases, emphasizing the intricate interaction between the biological clock and risk factors. There are novel genetic associations between the *NR1D1* and *NR1D2* clock gene and carbohydrate and lipid metabolism-related diseases. The rs4858095 SNP from *NR1D2* in male but not in female is associated with increased risk of dyslipidemia [33]. Furthermore, our recent study demonstrated that the *NR1D1* rs2314339 and rs72836608 SNPs are associated with increased CAD risk particularly in male patients, and are potentially influenced by factors such as age [34]. This framework helps contextualize the phenotypic difference observed between the two p.I148V carriers in our cohort. Patient 1 was a 41-year-old male who was overweight and had been diagnosed with dyslipidemia. He required treatment with insulin and metformin. He was also diagnosed with Hashimoto’s thyroiditis. Conversely, Patient 2, a 23-year-old female carrying the same variant, adequate glycemic control and a normal lipid profile. These contrasting metabolic patterns may reflect differential *NR1D2* expression across sex and age groups, consistent with reports that *NR1D2*-mediated transcriptional activity is more pronounced in males and increases with age [32]. Therefore, while p.I148V is unlikely to be a mutation causing a fully penetrant disease on its own, its metabolic effect may be modulated by systemic factors such as circadian regulation, endocrine environment, and age-related transcriptional changes. Patient 3, who carries the p.R286W variant, exhibited a clinical phenotype consistent with the heterogeneous nature of MODY. The patient’s phenotype was characterized by isolated elevations in LDL-C, hypertension, and mild hyperglycemia. In addition, this patient was diagnosed with Hashimoto’s thyroiditis. These features are more commonly associated with polygenic metabolic profiles than with classic monogenic diabetes. In silico prediction profile for p.R286W, which predominantly includes benign or borderline scores, suggests that its functional impact is likely modest or context-dependent.

Of particular significance is the observation that *NR1D2* is located on chromosome 3, within the same nuclear receptor gene cluster as thyroid hormone receptor-β (THRB). This finding lends further support to the hypothesis that there is an evolutionarily conserved relationship between NR1D2 and thyroid hormone signaling pathways [35]. Notwithstanding this genomic linkage, patients in our cohort predominantly demonstrated preserved thyroid function and a low-grade autoantibody positivity, which is consistent with background population rates as opposed to NR1D2-specific dysfunction. Consequently, the extant clinical data do not indicate that the p.R286W variant has a measurable effect on thyroid physiology, and the autoimmune thyroid markers observed in patients 1 and 3 can be more reasonably explained by a common autoimmune predisposition rather than the effects of the *NR1D2* variant. When considered collectively, these findings suggest that *NR1D2* variants may not directly cause monogenic diabetes but instead contribute to the modulation of metabolic phenotypes, particularly lipid-related traits. This interpretation is consistent with evidence from MODY cohorts, which demonstrates that rare variants in non-canonical genes frequently function as metabolic modifiers rather than as primary etiologic mutations [36, 37].

The observed heterogeneity across the three cases in this study supports a model in which *NR1D2* variation interacts with circadian–metabolic pathways, sex- and age-dependent expression patterns, and the underlying polygenic background to shape individual metabolic profiles. The clinical features exhibited by the three carriers of the *NR1D2* variant further substantiate this perspective and are in alignment with mechanistic insights derived from contemporary circadian metabolic research. *NR1D2*, in conjunction with its paralog *NR1D1*, constitutes a fundamental element of the molecular circadian clock, regulating a variety of metabolic processes, including lipid metabolism, adipocyte differentiation, skeletal muscle energy expenditure, and mitochondrial dynamics [38]. Despite the ambiguity surrounding the precise functional consequences of *NR1D2* variation in humans, experimental studies have demonstrated that alterations in *NR1D2* expression influence insulin secretion, glucose handling, and energy balance [11]. The pathophysiology of MODY is fundamentally characterized by impaired β-cell function and dysregulated insulin secretion [39]. In this context, *NR1D2* emerges as a biologically plausible modifier locus that may contribute to the observed phenotypic variation in MODY.

This study has some important limitations. Firstly, the study was conducted retrospectively, and the probands were distributed across different regions of Türkiye, which hindered the collection of DNA samples from their family members. In addition, several affected relatives had already passed away before the initiation of genetic analyses. As a result, genetic analyses for *NR1D2* could not be performed on affected family members. Despite these constraints, pedigree analyses revealed diabetes across three consecutive generations in all three families, providing supportive evidence for a hereditary component compatible with an autosomal dominant inheritance pattern (Fig. 1). Secondly, the non-intronic regulatory regions of *NR1D2*, encompassing the promoter and potential enhancer elements, were not subjected to sequencing. The genetic analysis was based on a targeted next-generation sequencing panel that exclusively covered coding exons and exon–intron boundaries. This approach precluded the evaluation of upstream regulatory variants that may influence NR1D2 expression or activity. Subsequent investigations that employ whole-genome sequencing or promoter-focused assays will be invaluable in determining whether regulatory variants contribute to the phenotypic spectrum observed in these families. Thirdly, comprehensive lipidomic profiling could not be performed. Due to the retrospective nature of the study, only conventional clinical fasting lipid parameters (triglycerides, LDL-C, and HDL-C) were available. These parameters, however, do not encompass the comprehensive lipidomic landscape pertinent to NR1D2-mediated metabolic regulation. Therefore, the potential associations between *NR1D2* variation and detailed lipidomic signatures remained to be elucidated. Subsequent prospective studies incorporating untargeted or targeted lipidomics will be necessary to determine whether these *NR1D2* mutations influence specific lipid pathways. Given that *NR1D2* functions as an integral component of the molecular circadian clock, alterations in circadian hormonal rhythms may be relevant to the metabolic features observed in our patients. Prior studies have demonstrated that disrupted nocturnal melatonin secretion is associated with immunometabolic dysregulation in humans, including T2DM and ischemic stroke patients [15, 40, 41]. Although melatonin rhythms could not be assessed in our cohort, these findings highlight the broader physiological context in which *NR1D2* variation may operate.

## Conclusion

Recent evidence has challenged the traditional view of MODY as a condition explained solely by high-penetrance monogenic mutations, particularly as increasing numbers of low-effect or context-dependent variants have been identified that do not uniformly produce a classical monogenic phenotype [28]. This has led to the hypothesis that certain rare variants may act as metabolic modifiers, influencing disease expression without functioning as primary causal alleles. Within this framework, examining the *NR1D2* variants identified in three MODY-X patients—together with their clinical profiles and comparison to other diabetes subtypes—provides an opportunity to explore potential interactions between circadian regulation and metabolic homeostasis. However, the retrospective design of the study precluded the assessment of circadian rhythm parameters, including sleep–wake patterns or diurnal hormonal measurements, which could have provided additional mechanistic insight. Future prospective studies will incorporate systematic evaluation of family members and employ in vitro and in vivo functional assays to clarify the biological impact of these missense variants and their potential role as modifiers within circadian–metabolic pathways.

## Supplementary Information

Below is the link to the electronic supplementary material.

Supplementary Material 1

Supplementary Material 2
