# Supplementary material for: Evaluation of rare NR1D2 variants in MODY-X: clinical, genetic, and in silico insights
Source: J Endocrinol Invest. 2026 Feb 6;49(6):1421–31. doi: 10.1007/s40618-026-02821-7 (PMC13219095; doi:10.1007/s40618-026-02821-7)
Supplement: Supplementary file 1 — Supplementary Material 1 [file 40618_2026_2821_MOESM1_ESM.doc]

Dear Editor,

We are sincerely grateful to you and the reviewers for the detailed and constructive comments provided on our manuscript (Manuscript ID: JENI-D-25-01372), originally entitled “*Could Circadian Rhythm Transcription Factor NR1D2 Be a New Candidate MODY Gene?*” and now revised as “*Evaluation of Rare NR1D2 Variants in MODY-X: Clinical, Genetic, and In Silico Insights*.” All reviewer suggestions have been thoroughly addressed, and corresponding revisions have been incorporated into the manuscript to strengthen its scientific contribution.

All newly added analyses, rewritten sections, and clarifications have been highlighted in yellow in the main text to facilitate evaluation. We believe that these revisions substantially improve the manuscript by enhancing the presentation of clinical data, refining the interpretation of the NR1D2 variants, expanding the discussion on their potential metabolic and circadian relevance, and more clearly articulating the methodological limitations.

In addition, the revised manuscript has become considerably longer due to the inclusion of new tables, extended analyses, and the expanded Discussion section requested by the reviewers. Therefore, we kindly request that our submission be reclassified from “*Rapid Communication*” to “*Original Article*,” as this format is now more appropriate for the scope and depth of the work.

Furthermore, an additional co-author, Dr. Ummu Mutlu, has been included in the revised manuscript due to their substantial contributions to the data analysis and manuscript revision.

We hope that the revised version satisfactorily addresses all reviewer comments and enhances the clarity, rigor, and overall quality of the manuscript.

We look forward to hearing from you at your earliest convenience.

Kind regards,

**Professor Ilhan Satman, MD**
Corresponding Author on behalf of all co-authors

**Reviewer comments:**

**Reviewer #1:**

The authors have investigated potentially novel mutations in NR1D2 gene that may be Associated with maturity-onset diabetes of the young that do not have a known casual variant (MODY-X). Authors have identified two rare missense mutations (Ile148Val in exon 4 and Arg286Trp in exon 5) by in silico prediction tools. The authors propose that these NR1D2 variants may represent novel causative mutations for MODY in the Turkish population, though further studies are needed to confirm if they are indeed pathogenic.

**Q1.** I ask the authors to include tables for the MODY-X patients tested (with or without the mentioned *NR1D2* mutations). Also, a table focusing on detailed data of the 3 patients studied in this manuscript.

**Response 1.** We are grateful for your valuable suggestion. In response, a new table (Table 1) has been included, which summarizes the demographic and clinical characteristics of the group, including all MODY-X patients (with or without the mentioned *NR1D2* mutations). Furthermore, a distinct table (Table 3) provides detailed clinical, biochemical, and genetic data for the three *NR1D2*-positive cases examined in this study.

This issue is addressed in the following sections of the article:

***In Results Section***

**Clinical and Metabolic Characteristics of the MODY-X Cohort**

The MODY-X cohort exhibited clinical features consistent with early-onset, non-insulin-dependent diabetes. The mean age of the patients and the mean age at diabetes onset were 31.6 ± 19.3 years and 19.8 ± 11.7 years, respectively. All MODY-X patients exhibited a positive family history of diabetes, indicative of a typical strong genetic background. Anthropometric and metabolic parameters exhibited substantial variability among MODY-X patients. The median body mass index (BMI) was 25.6 kg/m², indicating that a proportion of cases exhibited mild degrees of overweight. The markers of glycemic control exhibited significant increase (mean fasting plasma glucose (FPG) 165.7 ± 70.5 mg/dL and glycosylated hemoglobin A1c (HbA1c) (%) 7.48 ± 1.79%). The study found that 36.8% (n=7) of the patient population exhibited a HOMA-IR score above the reference value of 2.5, indicative of insulin resistance. The lipid profile exhibited a heterogeneous distribution, manifesting a dyslipidemic pattern in some patients. Thyroid function tests and autoantibodies were largely within reference limits. Liver and kidney functions were preserved in most cases, and diabetes-related complications were relatively low (Table 1).

**Clinical and Metabolic Characteristics of NR1D2 Variant Carriers**

**Patient 1** (male, 41-year-old) carried the *NR1D2* p.I148V mutation. He was diagnosed with early-onset diabetes (FPG 100 mg/dL, HbA1c 8.5%). The BMI was overweight (28.5 kg/m²). The patient's lipid profile revealed a hypertriglyceridemia (448 mg/dL) and low HDL-C (33 mg/dL), indicating dyslipidemia. The elevated thyroid autoantibodies against thyroid peroxidase and thyroglobulin (anti-TPO: 55.6 IU/mL; anti-Tg: 261 IU/mL) were identified, while thyroid function was within normal ranges. The kidney and liver parameters were within reference limits.

**Patient 2** (female, 23-year-old) also carried the p.I148V mutation. The patient first presented with prediabetes (FPG: 110 mg/dL and HbA1c: 5.7%), soon after turned to mild diabetes accompanied by an elevated BMI of 26.7 kg/m². In contrast to Patient 1, Patient 2 exhibited a normal fasting lipid profile and no autoimmune thyroiditis.

**Patient 3** (male, 58-year-old) with the *NR1D2* p.R286W mutation presented with moderate hyperglycemia (FPG 144 mg/dL, HbA1c 7.3%) and hypertension. The BMI was normal (23.9 kg/m²). While fasting triglycerides was within normal limits, low-density lipoprotein cholesterol (LDL-C) was moderately elevated (151 mg/dL) despite the administration of statin therapy. Serum anti-Tg was high, yet thyroid hormones were within the normal range.

During follow-up, none of these patients developed any microvascular complications. All three maintained normal β-cell reserve. In Patient 1 and Patient 3, lipid levels remained high despite anti-lipid therapy. The thyroid ultrasound images revealed heterogeneous parenchymal tissue, and a diagnosis of Hashimoto's thyroiditis was made. However, as thyroid hormone levels remained normal, they did not require L-thyroxine replacement therapy. Clinical and laboratory data of patients with *NR1D2* mutations detected at the time of genetic testing are shown in Table 3.

***In Discussion section***

….

Increasing evidence suggests that circadian dysfunction is associated with increased insulin resistance and metabolic irregularities [31]. The nuclear receptor NR1D2 has emerged as a promising therapeutic target due to its critical involvement in various biological processes, including circadian rhythms, inflammation, tumor formation, and glucose and lipid metabolism [32]. However, further research is necessary to elucidate the underlying mechanisms. Since NR1D1 and NR1D2 exhibit overlapping tissue distribution and partially redundant transcription functions, abnormalities in one paralog may alter compensatory mechanisms of the other. Importantly, *NR1D1/NR1D2* expression is known to increase in an age-dependent and male-specific manner [33], suggesting that metabolic traits may be differentially modulated by these transcription factors depending on sex and age. Clock gene polymorphisms exhibit associations with clinical markers of noncommunicable diseases, emphasizing the intricate interaction between the biological clock and risk factors. There are novel genetic associations between the *NR1D1* and *NR1D2* clock gene and carbohydrate and lipid metabolism-related diseases. The rs4858095 SNP from *NR1D2* in male but not in female is associated with increased risk of dyslipidemia [34]. Furthermore, our recent study demonstrated that the *NR1D1* rs2314339 and rs72836608 SNPs are associated with increased CAD risk particularly in male patients, and are potentially influenced by factors such as age [35]. This framework helps contextualize the phenotypic difference observed between the two p.I148V carriers in our cohort. Patient 1 was a 41-year-old male who was overweight and had been diagnosed with dyslipidemia. He required treatment with insulin and metformin. He was also diagnosed with Hashimoto's thyroiditis. Conversely, Patient 2, a 23-year-old female carrying the same variant, adequate glycemic control and a normal lipid profile. These contrasting metabolic patterns may reflect differential *NR1D2* expression across sex and age groups, consistent with reports that *NR1D2*-mediated transcriptional activity is more pronounced in males and increases with age [33]. Therefore, while p.I148V is unlikely to be a mutation causing a fully penetrant disease on its own, its metabolic effect may be modulated by systemic factors such as circadian regulation, endocrine environment, and age-related transcriptional changes. Patient 3, who carries the p.R286W variant, exhibited a clinical phenotype consistent with the heterogeneous nature of MODY. The patient's phenotype was characterized by isolated elevations in LDL-C, hypertension, and mild hyperglycemia. In addition, this patient was diagnosed with Hashimoto's thyroiditis. These features are more commonly associated with polygenic metabolic profiles than with classic monogenic diabetes. *In silico* prediction profile for p.R286W, which predominantly includes benign or borderline scores, suggests that its functional impact is likely modest or context-dependent.

Of particular significance is the observation that *NR1D2* is located on chromosome 3, within the same nuclear receptor gene cluster as thyroid hormone receptor-β (THRB). This finding lends further support to the hypothesis that there is an evolutionarily conserved relationship between NR1D2 and thyroid hormone signaling pathways [36]. Notwithstanding this genomic linkage, patients in our cohort predominantly demonstrated preserved thyroid function and a low-grade autoantibody positivity, which is consistent with background population rates as opposed to NR1D2-specific dysfunction. Consequently, the extant clinical data do not indicate that the p.R286W variant has a measurable effect on thyroid physiology, and the autoimmune thyroid markers observed in patients 1 and 3 can be more reasonably explained by a common autoimmune predisposition rather than the effects of the *NR1D2* variant. When considered collectively, these findings suggest that *NR1D2* variants may not directly cause monogenic diabetes but instead contribute to the modulation of metabolic phenotypes, particularly lipid-related traits. This interpretation is consistent with evidence from MODY cohorts, which demonstrates that rare variants in non-canonical genes frequently function as metabolic modifiers rather than as primary etiologic mutations [37,38].

The observed heterogeneity across the three cases in this study supports a model in which *NR1D2* variation interacts with circadian–metabolic pathways, sex- and age-dependent expression patterns, and the underlying polygenic background to shape individual metabolic profiles. The clinical features exhibited by the three carriers of the *NR1D2* variant further substantiate this perspective and are in alignment with mechanistic insights derived from contemporary circadian metabolic research. *NR1D2*, in conjunction with its paralog *NR1D1*, constitutes a fundamental element of the molecular circadian clock, regulating a variety of metabolic processes, including lipid metabolism, adipocyte differentiation, skeletal muscle energy expenditure, and mitochondrial dynamics [39]. Despite the ambiguity surrounding the precise functional consequences of *NR1D2* variation in humans, experimental studies have demonstrated that alterations in *NR1D2* expression influence insulin secretion, glucose handling, and energy balance [11]. The pathophysiology of MODY is fundamentally characterized by impaired β-cell function and dysregulated insulin secretion [40]. In this context, *NR1D2* emerges as a biologically plausible modifier locus that may contribute to the observed phenotypic variation in MODY.

**Table 1. Characteristics of the MODY-X group**

|  | **n** | **%** | **Reference values** |
| --- | --- | --- | --- |
| **Demographic** |  |  |  |
| Gender (female/male) | 12 / 7 | 63 / 37 |  |
| Family history of diabetes (yes/no) | 19 / 0 | 100 / 0 |  |
|  | **X± SD** | **Median (IQR)** |  |
| Age (year) | 31.6 ± 19.3 | 41 (14.5) |  |
| Age-at-onset of diabetes (year) | 19.8 ± 11.7 | 24 (15.5) |  |
| Body mass index at diagnosis (kg/m^2^) | 25.6 ± 5.9 | 28.5 (3.5) | <25 |
| Systolic blood pressure (mmHg) | 118.5 ± 5.5 | 120 (7.5) | <120 |
| Diastolic blood pressure (mmHg) | 74.7 ± 8.6 | 80 (5) | <80 |
| **Glycemic markers** |  |  |  |
| Fasting plasma glucose (mg/dL) | 165.7 ± 70.5 | 196 (99) | 70 – 100 |
| Glycosylated hemoglobin A1c (%) | 7.48 ± 1.79 | 8.5 (3.1) | 4 – 5.6 |
| C-peptide (ng/mL) | 1.94 ± 0.90 | 1.17 (1.34) | 1.1 – 4.4 |
| HOMA-IR | 4.78 ± 1.58 | 1.57 (1.45) | <2.5 |
| **Lipid profile** |  |  |  |
| Triglycerides (mg/dL) | 135.5 ± 99.1 | 157 (282.5) | <150 |
| High-density lipoprotein-cholesterol (mg/dL) | 47.5 ± 15.5 | 43 (23.5) | >40 |
| Low-density lipoprotein-cholesterol (mg/dL) | 109.1 ± 31.7 | 120 (29.5) | <130 |
| **Thyroid function** |  |  |  |
| Thyroid stimulating hormone (mIU/L) | 2.37 ± 1.35 | 1.68 (3.04) | 0.27 – 4.2 |
| Free thyroxine (ng/dL) | 15.79 ± 2.06 | 16. 08 (1.02) | 12 – 22 |
| Anti-thyroid peroxidase antibody (IU/mL) | 12.9 ± 12.7 | 9.0 (30.8) | <35 |
| Anti-thyroglobulin antibody (IU/mL) | 67.3 ± 53.5 | 25.8 (137.5) | <115 |
| **Liver function** |  |  |  |
| Alanine aminotransaminase (U/L) | 30.6 ± 20.7 | 22 (41) | 5 – 45 |
| Aspartate aminotransaminase (U/L) | 24.6 ± 12.9 | 40 (26) | 5 – 42 |
| Gamma glutamyl transferase (U/L) | 39.1 ± 31.9 | 69 (63) | 5 – 85 |
| High-sensitivity C-reactive protein (mg/L) | 2.91 ± 2.80 | 1.01 (5.08) | <5 |
| **Kidney function** |  |  |  |
| Uric acid (mg/dL) | 4.2 ± 1.5 | 4.2 (3.4) | 2.5 – 7.5 |
| Blood urea nitrogen (mg/dL) | 14.3 ± 6.4 | 15 (10.5) | 8 – 22 |
| Urea (mg/dL) | 25.7 ± 11.3 | 15 (26.5) | 0 – 50 |
| Creatinine (mg/dL) | 0.71 ± 0.21 | 0.8 (0.32) | 0.7 – 1.4 |
| Estimated glomerular filtration rate (mL/min./1.73 m^2^) | 109.2 ± 31.6 | 110 (33.5) | >90 |
| Urine albumin-to-creatinine ratio (mg/g) | 13.3 ± 6.7 | 3.3 (6.1) | <30 |
| **Diabetes-related complications** | **n** | **%** |  |
| Nephropathy (yes/no) | 2 / 17 | 10.5 / 89.5 |  |
| Neuropathy (yes/no) | 4 / 15 | 21.1 / 78.9 |  |
| Retinopathy (yes/no) | 2 / 17 | 10.5 / 89.5 |  |

Categorical variables were presented as sample size (n) and percentage (%), while continuous variables were presented as mean (± standard deviation) and median (interquartile range).

MODY-X, maturity-onset diabetes of the young patients with no confirmed genetic causes; HOMA-IR, homeostatic model assessment of insulin resistance.

**Table 3.** The clinical characteristics of MODY-X patients with *NR1D2* gene mutation

|  | **Patient 1** | **Patient 2** | **Patient 3** |
| --- | --- | --- | --- |
| Patient code | DAH1 | DAH2 | DAH56 |
| *NR1D2* mutation | p.Ile148Val (rs148928938) | p.Ile148Val (rs148928938) | p.Arg286Trp (rs768518229) |
| **Demographic** |  |  |  |
| Gender | Male | Female | Male |
| Age (year) | 41 | 23 | 58 |
| Age at diagnosis of diabetes (year) | 34 | 16 | 38 |
| Body mass index at diagnosis (kg/m^2^) | 28.5 | 26.7 | 23.9 |
| **Glycemic markers** |  |  |  |
| Fasting plasma glucose (mg/dL) | 100 | 110 | 144 |
| Glycosylated hemoglobin A1c (%) | 8.5 | 5.7 | 7.3 |
| C-peptide (ng/mL) | 1.99 | 1.69 | 1.91 |
| HOMA-IR | 1.57 | 1.57 | 1.60 |
| **Lipid profile** |  |  |  |
| Triglycerides (mg/dL) | 448 | 115 | 91 |
| High-density lipoprotein-cholesterol (mg/dL) | 33 | 59 | 43 |
| Low-density lipoprotein-cholesterol (mg/dL) | 128 | 127 | 151 |
| **Thyroid function** |  |  |  |
| Thyroid stimulating hormone (mIU/L) | 2.07 | 1.45 | 3.54 |
| Free thyroxine (ng/dL) | 16.4 | 11.7 | 14.7 |
| Anti-thyroid peroxidase antibody (IU/mL) | 55.6 | 7 | 9.6 |
| Anti-thyroglobulin antibody (IU/mL) | 261 | 15 | 223 |
| **Liver function** |  |  |  |
| Alanine aminotransaminase (U/L) | 19 | 9 | 18 |
| Aspartate aminotransaminase (U/L) | 14 | 10 | 21 |
| Gamma glutamyl transferase (U/L) | 18 | 4 | 22 |
| High-sensitivity C-reactive protein (mg/L) | 0.40 | 1.28 | 3.57 |
| **Kidney function** |  |  |  |
| Uric acid (mg/dL) | 5.3 | 1.3 | 4.2 |
| Blood urea nitrogen (mg/dL) | 15 | 3 | 21 |
| Urea (mg/dL) | 15 | 5 | 42 |
| Creatinine (mg/dL) | 0.8 | 0.7 | 0.8 |
| Estimated glomerular filtration rate (mL/min./1.73 m^2^) | 110 | 161 | 98.5 |
| Urine albumin-to-creatinine ratio (mg/g) | 12.8 | 19 | 8.27 |
| **Diabetes-related complications** |  |  |  |
| Nephropathy (Yes/No) | No | No | No |
| Neuropathy (Yes/No) | No | No | No |
| Retinopathy (Yes/No) | No | No | No |
| Other chronic diseases | Hashimoto thyroiditis | No | Hypercholesterolemia,  Hashimoto thyroiditis, Hypertension |
| **Medications** | Insulin, Metformin | Insulin | SU, Insulin, Statin, ARB, Thiazide |

HOMA-IR, homeostatic model assessment of insulin resistance; SU, sulfonylurea; ARB, angiotensin-II receptor blocker.

**Q2.** Have the authors considered observing the mutation observing patterns in affected family members.

**Response 2:** We sincerely thank the reviewer for this insightful comment emphasizing the importance of familial segregation analysis. We fully agree that confirming the presence or absence of the identified NR1D2 variants in affected relatives would provide stronger genetic evidence for pathogenicity.

As we stated in the Research Design and Methods section, in the current investigation we used genetic material obtained from our previous study (MODY-Ist)., and the majority of the probands resided in disparate regions of Türkiye, which made it logistically challenging to obtain biological samples from their family members. Furthermore, it is also crucial to note that a number of affected relatives had unfortunately passed away prior to the commencement of our genetic investigation. Therefore, family-based segregation analyses could not be performed at this stage.

These limitations have been clearly delineated in the Discussion section, and it has been emphasized that prospective follow-up studies are planned to include extended family genotyping once additional samples can be collected.

This issue is addressed in the following sections of the article:

***In Results section***

**Pedigree analyses of the three *NR1D2* mutation–positive patients.** Pedigree evaluation revealed multigenerational transmission of diabetes in all *NR1D2* variant–positive families (Figure 1), reflecting a pattern concordant with autosomal dominant inheritance. Despite the inability to conduct segregation analysis due to the retrospective study design and the unavailability of family genetic samples, the recurrence of diabetes across multiple generations offers compelling evidence supporting a shared genetic predisposition among these relatives.

**Fig.1** The pedigree structures of three MODY-X probands carrying the NR1D2 missense variants. The probands are indicated by arrows. In all families, diabetes shows vertical transmission across at least three generations, consistent with an autosomal dominant inheritance pattern. Genetic testing was performed only in the probands; therefore, the NR1D2 variant status of affected relatives remains unknown.

***In Discussion section***

……

This study has some important limitations. Firstly, the study was conducted retrospectively, and the probands were distributed across different regions of Türkiye, which hindered the collection of DNA samples from their family members. In addition, several affected relatives had already passed away before the initiation of genetic analyses. As a result, genetic analyses for *NR1D2* could not be performed on affected family members. Despite these constraints, pedigree analyses revealed diabetes across three consecutive generations in all three families, providing supportive evidence for a hereditary component compatible with an autosomal dominant inheritance pattern (Figure 1).

**Q3.** Have the authors also consider sequencing the non-intronic regulatory regions as well as the promoter region of the gene.

**Response 3:** In the present study, only the coding exons and exon–intron boundaries of *NR1D2* were sequenced, as the analysis was based on a targeted next-generation sequencing (NGS) panel that did not cover upstream regulatory or promoter regions. Consequently, potential pathogenic variants within non-coding regulatory elements, such as promoters, enhancers, or untranslated regions (UTRs), could not be assessed. We acknowledge this as a limitation and have clarified it in the revised Discussion section. However, we fully agree that regulatory variants may significantly influence *NR1D2* expression or transcriptional regulation, and future whole-genome or targeted promoter sequencing studies are warranted to address this important aspect.

This issue is addressed in the following sections of the article:

***In Discussion section***

…

Secondly, the non-intronic regulatory regions of *NR1D2*, encompassing the promoter and potential enhancer elements, were not subjected to sequencing. The genetic analysis was based on a targeted next-generation sequencing panel that exclusively covered coding exons and exon–intron boundaries. This approach precluded the evaluation of upstream regulatory variants that may influence NR1D2 expression or activity. Subsequent investigations that employ whole-genome sequencing or promoter-focused assays will be invaluable in determining whether regulatory variants contribute to the phenotypic spectrum observed in these families.

**Q4.** I ask the authors to elaborate more on how confident they are in calling them "causative variants" in a monogenic disease.

**Response 4:** We thank the reviewer for raising this important point. We fully acknowledge that the currently available evidence does not allow us to classify the NR1D2 variants identified in our patients as definitively “causative” in the context of monogenic diabetes. Although both variants are extremely rare and segregate within families exhibiting multigenerational diabetes, the interpretation necessarily relies on clinical features, pedigree information, and in silico predictions rather than functional validation or segregation analyses. Moreover, the in silico tools generated heterogeneous outputs, a pattern that supports cautious interpretation regarding their functional impact. For these reasons, we avoid referring to the variants as “causative” in the revised manuscript. Instead, we describe them as rare variants within a biologically plausible modifier locus (NR1D2) that may contribute to metabolic phenotypes but cannot be established as primary etiologic mutations.

**Q5.** Have the authors considered doing functional assays to see in vitro or in vivo how these mutations affect the protein function.

**Response 5.** We sincerely thank the reviewer for this important comment. We fully agree that functional *in vitro* or *in vivo* assays would provide the most definitive evidence regarding the biological impact of the *NR1D2* variants. However, such experiments could not be performed in the present study because it was conducted retrospectively and biological material beyond routine clinical samples was not available for mechanistic assays.

To address this limitation, we performed a comprehensive multi-platform *in silico* evaluation using SIFT, PolyPhen-2, MutationTaster2025, FATHMM-XF, REVEL, CADD, and DynaMut2. We would like to emphasize that several of the tools we used specifically assess the potential impact of the variants on protein function and structural integrity. In particular, PolyPhen-2, SIFT, and FATHMM-XF directly predict functional consequences of amino acid substitutions, while DynaMut2 evaluates the effect of each missense variant on protein stability (ΔΔG) using structural modeling. REVEL and CADD further provide ensemble and integrative metrics that incorporate protein-level features among other biological parameters. Although these approaches do not replace *in vitro* or *in vivo* functional assays, they provide protein-focused computational evidence that partially compensates for the lack of experimental validation in this retrospective study.

These *in silico* tools yielded heterogeneous predictions—a pattern typical of variants with modest or context-dependent effects rather than high-penetrance monogenic pathogenicity. Taken together, the *in silico* findings support a low-to-moderate effect model, suggesting that these *NR1D2* variants may act as metabolic modifiers rather than definitive causative mutations.

This issue is addressed in the following sections of the article (furthermore, these two variants are illustrated in Figure 2):

***In Research Design and Methods - Bioinformatic Analysis section***

**…**

To assess protein-level functional and structure consequences, SIFT (Sorting Intolerant From Tolerant) [20], Polyphen-2 (Polymorphism Phenotyping v2) [21], FATHMM-XF [22], and DynaMut2 [23] were employed, as these tools directly model the potential impact of amino acid substitutions on protein function or stability. Additionally, broader pathogenicity predictors including MutationT@ster2025 [24], REVEL (Rare Exome Variant Ensemble Learner) [25], and CADD (Combined Annotation Dependent Depletion) [26] tools were used to integrate evolutionary, biochemical, and multi-feature information. Each variant was also queried in the ClinVar database to determine previously reported clinical significance [27].

***In Results- In Silico Analysis section***

The potential functional impact of the *NR1D2* missense variants p.I148V and p.R286W was assessed using a comprehensive multi-platform *in silico* prediction strategy (Table 2). For p.I148V, several tools predicted a damaging or destabilizing effect. PolyPhen-2 indicated a likely damaging impact (0.954), SIFT classified the substitution as deleterious at the decision threshold (0.05), and MutationTaster2025 identified the variant as disease-causing. FATHMM-XF also supported a damaging effect (0.737876), while REVEL yielded a likely pathogenic score (0.661). The CADD score (23) placed the variant within the deleterious range, and DynaMut2 predicted a notable reduction in protein stability (ΔΔG = –1.76 kcal/mol). For p.R286W, the *in silico* predictions were more heterogeneous. PolyPhen-2 suggested a benign effect (0.014), and FATHMM-XF exhibited a similarly benign tendency (0.154967). The SIFT yielded a borderline tolerated value (0.06), whereas MutationTaster2025 classified the variant as disease-causing. The REVEL generated an indeterminate score (0.501), and the CADD value (20.6) fell near the lower end of the deleterious spectrum. The DynaMut2 model predicted only mild structural destabilization (ΔΔG = –0.58 kcal/mol). However, no pathogenicity data is available for these mutations in ClinVar. Taken together, these results indicate that p.I148V is supported by a more consistent deleterious signature across multiple prediction algorithms, whereas p.R286W exhibits a mixed profile with predominantly benign or borderline outputs. This pattern is consistent with a potential low-to-moderate effect model rather than a uniformly pathogenic impact.

**Table 2.** In silico pathogenicity predictions for the *NR1D2* p.I148V and p.R286W variants across multiple computational platforms

| ***NR1D2* mutation (GRCh38.p14)** | ***NR1D2* p.Ile148Val** | | ***NR1D2* p.Arg286Trp** | |
| --- | --- | --- | --- | --- |
| Chromosomal position | chr3: 23959740 | | chr3: 23962315 | |
| Rs no | rs148928938 | | rs768518229 | |
| Reference allele | A | | C | |
| Minor allele | G | | T | |
| gnomAD Allele Frequency (v4.1.0) | G= 0.00007373 | | T= 0.000003098 | |
| Transcript variant | NM_001145425.2:c.217A>C | | NM_001145425.2:c.631C>T | |
| ***In Silico* Predictors** | **Score / Prediction** | **Interpretation** | **Score / Prediction** | **Interpretation** |
| Polyphen | 0.954 | Probably damaging | 0.014 | Benign |
| SIFT | 0.05 | Deleterious (at threshold) | 0.06 | Tolarated (borderline) |
| MutationT@ster2025 | Deleterious | Disease causing | Deleterious | Disease causing |
| FATHMM-XF | 0.737876 | Damaging | 0.154967 | Benign |
| REVEL | 0.661 | Likely pathogenic | 0.501 | Uncertain |
| CADD Phred | 23 | Deleterious | 20.6 | Deleterious |
| DynaMut2 (ΔΔG) | –1.76 kcal/mol | Destabilizing | –0.58 kcal/mol | Mild destabilizing |

***In Discussion section***

…

*In silico* analyses of the p.I148V and p.R286W variants yielded partially discordant results, thus illustrating the inherent limitations of relying on individual prediction tools for pathogenicity assessment. While multiple algorithms indicated deleterious or destabilizing effects for p.I148V, p.R286W showed predominantly benign or borderline outcomes. This heterogeneity is consistent with variants that exert modest or context-dependent effects, rather than the uniform signatures expected for high-penetrance monogenic mutations. While both variants are present in gnomAD at a low frequency, this finding alone does not substantiate a pathogenic role in the absence of segregation or functional data. These findings support the interpretation of the *NR1D2* variants as biologically plausible modifiers rather than definitive causative alleles.

**Fig.2** Structural localization of *NR1D2* missense variants p.I148V and p.R286W within the protein model. The model and residue-level visualizations were generated using ProtVar.

**Reviewer #2:** This is an interesting study reporting for the first time the association of 2 missense mutations in the NR1D2 gene in patients with MODY-X that do not have any potential genetic diagnosis. According the authors the 2 identified de novo mutations are qualified as of having allele frequencies are 74 per million and 3 per million so respectively of higher and very low frequencies. Many concerns should be addressed promptly by the authors:

**Q1:** The data should be presented and identified in a clear way rather than being added at the end of table showing patients and participants characteristics.

**Response 1:** We thank our reviewer for this valuable suggestion. In the revised manuscript, the genetic findings have been reinforced with additional *in silico* analyses and are now presented under a separate heading in the Results section. Furthermore, the tables have been restructured, and information on *NR1D2* mutations is now clearly separated from the general clinical characteristics of the MODY-X cohort.

**Q2.** As this protein has been already correlated with alterations in in circadian rhythms and carbohydrate and lipid metabolism, it would be interesting to see if any correlation with these mutations and the lipidomic profile of the patients?

**Response 2:** We appreciate the reviewer’s insightful comment. It is known that *NR1D2* plays a role in the circadian regulation of carbohydrate and lipid metabolism, and we agree that it is important to evaluate potential metabolic correlations. Since this study was retrospective, only routine clinical lipid measurements (triglycerides, LDL-C and HDL-C) were available. We analyzed these parameters specifically in three *NR1D2* variant carriers and included the relevant findings in the revised Results and Discussion sections. Since comprehensive lipidomic profiling was not performed in this cohort, broader lipidomic relationships could not be evaluated. This limitation is clearly stated in the article.

***In Discussion section***

**….**

**Thirdly, comprehensive lipidomic profiling could not be performed. Due to the retrospective nature of the study, only conventional clinical fasting lipid parameters (triglycerides, LDL-C, and HDL-C) were available. These parameters, however, do not encompass the comprehensive lipidomic landscape pertinent to NR1D2-mediated metabolic regulation. Consequently, the potential associations between *NR1D2* variation and detailed lipidomic signatures remained to be elucidated. Subsequent prospective studies incorporating untargeted or targeted lipidomics will be necessary to determine whether these *NR1D2* mutations influence specific lipid pathways.**

**Q3:** Since the gene NR1D2 is known as a clock gene, authors should confirm their data by checking the gene expression analysis at several time points and paralleling their data by measurement of the melatonin.

**Response 3:** We appreciate the reviewer’s insightful comment. While NR1D2 is indeed a core component of the molecular circadian clock, the present study was not designed as a chronobiology-focused investigation. Our primary aim was to examine coding-region variation in NR1D2 among MODY-X patients using genetic sequencing and in silico analyses. Detailed circadian phenotyping—such as time-series NR1D2 expression profiling, assessment of melatonin secretion rhythms, or controlled sampling across circadian phases—requires a prospective study framework with tightly timed biological collections and specialized laboratory protocols. These approaches are inherently incompatible with the retrospective clinical dataset analyzed here and therefore fall outside the methodological scope of the study.

Nevertheless, we agree that circadian mechanisms provide a biologically meaningful context for interpreting NR1D2 variation. As acknowledged in the Discussion section, prior human studies demonstrate that alterations in nocturnal melatonin secretion can be associated with immunometabolic dysregulation (Fiorina et al., Lancet, 1996; Fiorina et al., Scand J Immunol, 1999), suggesting that NR1D2-related effects may interact with broader circadian–metabolic pathways. Although circadian biomarkers could not be evaluated in our cohort, we have now expanded the Discussion to reflect this conceptual framework.

Future mechanistic studies specifically designed to investigate NR1D2’s chronobiological effects, including time-series sampling and circadian-regulated hormone measurements will be valuable for determining whether these variants modulate circadian–metabolic interactions.

**Q4:** I ask the author to consider citing the following papers:

Impaired nocturnal melatonin excretion and changes of immunological status in ischaemic stroke patients. Fiorina P, Lattuada G, Ponari O, Silvestrini C, DallAglio P.Lancet. 1996 Mar 9;347(9002):692-3. doi: 10.1016/s0140-6736(96)91246-5.PMID: 8596407.

Disruption of nocturnal melatonin rhythm and immunological involvement in ischaemic stroke patients. Fiorina P, Lattuada G, Silvestrini C, Ponari O, Dall'Aglio P.Scand J Immunol. 1999 Aug;50(2):228-31. doi: 10.1046/j.1365-3083.1999.00579.x.PMID: 10447930.

**Response 4:** We thank the reviewer for these valuable references. Both suggested papers (Fiorina et al., Lancet, 1996; Fiorina et al., Scandinavian Journal of Immunology, 1999), which report disruptions in nocturnal melatonin rhythms and associated immunological alterations, have now been cited in the Discussion to provide broader circadian and neuroendocrine context relevant to the role of NR1D2 as a clock gene.

***In Discussion section***

….

Given that NR1D2 functions as an integral component of the molecular circadian clock, alterations in circadian hormonal rhythms may be relevant to the metabolic features observed in our patients. Prior studies have demonstrated that disrupted nocturnal melatonin secretion is associated with immunometabolic dysregulation in humans, including T2DM and ischemic stroke patients [15, 41, 42]. Although melatonin rhythms could not be assessed in our cohort, these findings highlight the broader physiological context in which NR1D2 variation may operate.
