# Supplementary material for: Evaluation of rare NR1D2 variants in MODY-X: clinical, genetic, and in silico insights
Source: J Endocrinol Invest. 2026 Feb 6;49(6):1421–31. doi: 10.1007/s40618-026-02821-7 (PMC13219095; doi:10.1007/s40618-026-02821-7)
Supplement: Supplementary file 2 — Supplementary Material 2 [file 40618_2026_2821_MOESM2_ESM.doc]

**ISTANBUL UNIVERSITY**

**ISTANBUL FACULTY OF MEDICINE**

**DEPARTMENT OF INTERNAL MEDICINE**

**DIVISION OF ENDOCRINOLOGY AND METABOLISM**

November 23rd, 2025

Dear Editor,

We are sincerely grateful to you and the reviewers for the detailed and constructive comments provided on our manuscript (Manuscript ID: JENI-D-25-01372), originally entitled “*Could Circadian Rhythm Transcription Factor NR1D2 Be a New Candidate MODY Gene?*” and now revised as “*Evaluation of Rare NR1D2 Variants in MODY-X: Clinical, Genetic, and In Silico Insights*.” All reviewer suggestions have been thoroughly addressed, and corresponding revisions have been incorporated into the manuscript to strengthen its scientific contribution.

All newly added analyses, rewritten sections, and clarifications have been highlighted in yellow in the main text to facilitate evaluation. We believe that these revisions substantially improve the manuscript by enhancing the presentation of clinical data, refining the interpretation of the NR1D2 variants, expanding the discussion on their potential metabolic and circadian relevance, and more clearly articulating the methodological limitations.

In addition, the revised manuscript has become considerably longer due to the inclusion of new tables, extended analyses, and the expanded Discussion section requested by the reviewers. Therefore, we kindly request that our submission be reclassified from “*Rapid Communication*” to “*Original Article*,” as this format is now more appropriate for the scope and depth of the work.

Furthermore, an additional co-author, Dr. Ummu Mutlu, has been included in the revised manuscript due to their substantial contributions to the data analysis and manuscript revision.

We hope that the revised version satisfactorily addresses all reviewer comments and enhances the clarity, rigor, and overall quality of the manuscript.

We look forward to hearing from you at your earliest convenience.

Kind regards,

Professor Ilhan SATMAN, MD

Istanbul University

Istanbul Faculty of Medicine

Department of Internal Medicine

Division of Endocrinology and Metabolism

Istanbul, Türkiye

Email: [satmandiabet@gmail.com](mailto:satmandiabet@gmail.com); [satman@istanbul.edu.tr](mailto:satman@istanbul.edu.tr)

Istanbul University, Istanbul Faculty of Medicine, Dpt. Internal Medicine, Turgut Ozal Millet Caddesi, Capa, 34093-Istanbul, Türkiye. Phone: +90 212 414 20 00/31312
